# Photophysical Properties of BADAN Revealed in the Study of GGBP Structural Transitions

**DOI:** 10.3390/ijms222011113

**Published:** 2021-10-15

**Authors:** Alexander V. Fonin, Sergey A. Silonov, Iuliia A. Antifeeva, Olga V. Stepanenko, Olesya V. Stepanenko, Anna S. Fefilova, Olga I. Povarova, Anastasia A. Gavrilova, Irina M. Kuznetsova, Konstantin K. Turoverov

**Affiliations:** 1 Laboratory of Structural Dynamics, Stability and Folding of Proteins, Institute of Cytology, Russian Academy of Sciences, 4 Tikhoretsky Ave., 194064 Saint Petersburg, Russia; silonovsa25@yandex.ru (S.A.S.); julgag@yandex.ru (I.A.A.); sov@incras.ru (O.V.S.); lvs@incras.ru (O.V.S.); a.fefilova@incras.ru (A.S.F.); olp@incras.ru (O.I.P.); asultanbekova@incras.ru (A.A.G.); kkt@incras.ru (K.K.T.); 2 Research Center for Molecular Mechanisms of Aging and Age-Related Diseases, Moscow Institute of Physics and Technology, 141700 Dolgoprudny, Russia

**Keywords:** BADAN (6-bromoacetyl-2-dimetylaminonaphtalene) spectroscopy, apo- and holo-forms of GGBP (D-glucose/D-galactose-binding protein), pathways of GGBP unfolding

## Abstract

The fluorescent dye BADAN (6-bromoacetyl-2-dimetylaminonaphtalene) is widely used in various fields of life sciences, however, the photophysical properties of BADAN are not fully understood. The study of the spectral properties of BADAN attached to a number of mutant forms of GGBP, as well as changes in its spectral characteristics during structural changes in proteins, allowed to shed light on the photophysical properties of BADAN. It was shown that spectral properties of BADAN are determined by at least one non-fluorescent and two fluorescent isomers with overlapping absorbing bands. It was found that BADAN fluorescence is determined by the unsolvated “PICT” (planar intramolecular charge transfer state) and solvated “TICT” (twisted intramolecular charge transfer state) excited states. While “TICT” state can be formed both as a result of the “PICT” state solvation and as a result of light absorption by the solvated ground state of the dye. BADAN fluorescence linked to GGBP/H152C apoform is quenched by Trp 183, but this effect is inhibited by glucose intercalation. New details of the changes in the spectral characteristics of BADAN during the unfolding of the protein apo and holoforms have been obtained.

## 1. Introduction

Thanks to high sensitivity, simplicity, low cost, and possibility to be used as an express test, fluorescence methods are widely used in the study and analysis of living systems. For different tasks, researchers have a wide range of fluorescent methods in their arsenal; namely, intrinsic fluorescence, fluorescent proteins, external fluorescent probes, dyes, and so on. The fluorescent thiol-reactive solvatochromic dyes BADAN (6-bromoacetyl-2-dimetylaminonaphtalene), PRODAN (2-dimethylamino-6-propionylnaphthalene), and LAURDAN (2-dimethylamino-6-lauroylnaphthalene) are widely used for the site-directed protein labeling. PRODAN and LAURDAN are known to be used in several membrane studies. Their photophysical properties have been extensively studied and the existence of several states has been established, of PRODAN and its analogs, in both the ground and excited states [1,2,3,4,5,6,7,8,9,10,11,12,13,14,15,16,17,18]. It was shown that the fluorescence characteristics of PRODAN in 1,4-dioxane are due to two forms of the dye in the ground and two forms of PRODAN in the excited state, which differ in the magnitude of the dipole moment and the conformation of the carbonyl group [19].

The transition of this dye molecule from the ground to the excited state is associated with a significant redistribution of the electron density and an increase in the BADAN dipole moment due to charge transfer from the amino group to the carbonyl group of the dye molecule. This determines the solvatochromism of BADAN and the dependence of the fluorescence characteristics of this dye on the properties of its microenvironment [20,21,22,23]. The sensitivity of BADAN characteristics to the properties of its microenvironment and the short linker region connecting the acetyl-dimethylnaphthalene part of BADAN [24] provide information on protein structural changes in the region of dye localization. This allows the use of BADAN fluorescence in a number of applications, in particular, as a signal of biosensor systems for glucose when it is linked to D-glucose/D-galactose-binding protein (GGBP) [25,26,27,28,29,30]. The active site of GGBP is located between two domains and is formed by polar and aromatic amino acid residues [31,32]. The interaction of GGBP with glucose and galactose is characterized by high binding affinity and a significant change in the mutual arrangement of protein domains, causing a transition from a predominantly “open” conformation of GGBP to a predominantly “closed” conformation [31,33,34,35]. Localization of BADAN in the region of the active site of this protein, when it is linked to Cys 152 of the mutant form GGBP/H152C, allows one to register structural changes in GGBP during its interaction with glucose by changing the fluorescent characteristics of the dye [25,26,29].

Previously, we showed that the GGBP/H152C unfolding induced by chemical denaturants guanidine hydrochloride (GdnHCl) and urea recorded by intrinsic fluorescence of protein tryptophan residues and BADAN fluorescence differ significantly [36,37]. These results may be due to the fact that the microenvironment of BADAN and GGBP tryptophan residues reflects the conformational changes in different regions of the protein. BADAN is localized between the GGBP domains in the active center of the protein, while four out of five GGBP tryptophan residues are located in the C-terminal domain of GGBP [38,39,40]. However, there is still no exhaustive explanation of this effect. Perhaps, it is caused by the lack of complete understanding of BADAN photophysical properties, despite its wide application in various fields of life science [20,21,24,41,42,43,44,45,46,47,48,49,50,51,52,53,54]. Thus, the work aimed to clarify BADAN photophysical characteristics and explain its spectral characteristics in different structural states of GGBP.

## 2. Results and Discussion

**Figure 1:**
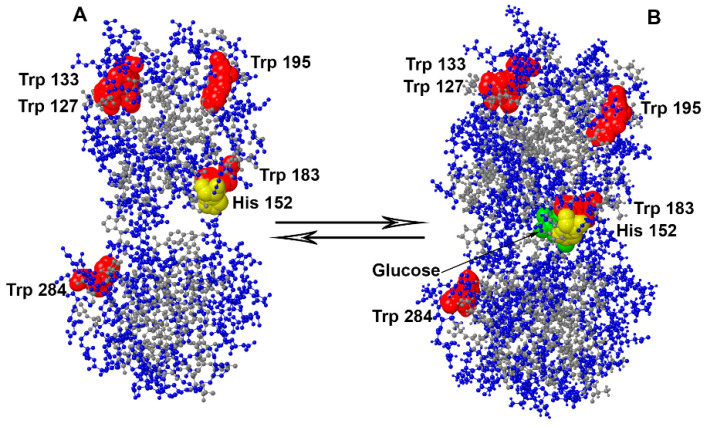
Spatial structure of D-glucose/D-galactose-binding protein (GGBP) in apoform panel (**A**) and holoform panel (**B**) according to X-ray structural analysis [33]. Polar GGBP residues are shown in blue; non-polar protein residues are in gray; GGBP tryptophan residues, four of five of which are located in the C-terminal domain of the protein, are shown in red; histidine residue 152 is shown in yellow (His152/Cys substitution provides linking of the fluorescent dye BADAN in the active center of GGBP); and the glucose molecule is shown in green.

In this work, we studied the spectral characteristics of BADAN linked to GGBP mutants (GGBP/H152C, GGBP/W284C, GGBP/H152C/W183F, and GGBP/H152C/W183A) in apo and holoform (Figure 1). In the mutant form GGBP/H152C, BADAN is located in close proximity to the glucose binding site. This form was proposed earlier as a sensitive element of a biosensor for glucose. In order to exclude the influence of tryptophan residue 183, also located in the glucose binding site, on the BADAN fluorescence, the variants GGBP/H152C/W183F and GGBP/H152C/W183A were studied. Finally, we examined the GGBP/W284C form, in which BADAN binds not to the glucose-binding center, but to the periphery of the protein.

### 2.1. Spectral Characteristics of BADAN Associated with Various Structural States of GGBP Variants

#### 2.1.1. GGBP/H152C Apo- and Holoforms

The fluorescence spectrum of BADAN attached to the GGBP/H152C apoform (λmax = 540 nm) is shifted towards longer wavelengths compared with the position of the spectrum of the free dye in aqueous solutions (2% dimethylformamamide/98% water) determined in [29]. As free BADAN is very poorly soluble in water [29], this result is not surprising and only indicates a high polarity of the environment of the dye linked to the protein. The relatively high fluorescence anisotropy of BADAN attached to GGBP/H152C in the absence of a ligand (r = 0.19, Table S1) suggests that the dye microenvironment is tightly packed. This is consistent with the spatial structure of the protein [55].

**Figure 2:**
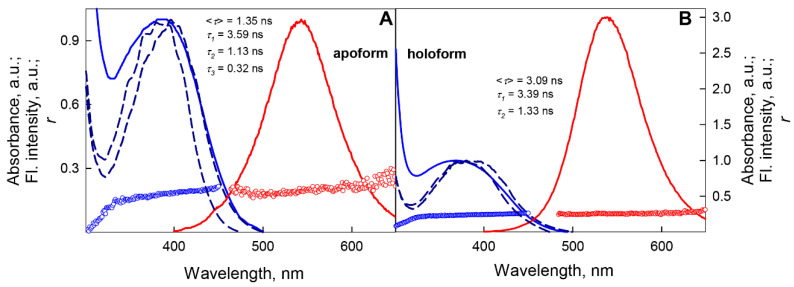
Spectral characteristics of BADAN bound to GGBP/H152C in apo panel (**A**) and holoform panel (**B**). The absorption and fluorescence spectra of BADAN are shown by solid blue and red curves, respectively. The absorption spectra are normalized to the signal intensity at the maximum of the long-wavelength band. The dye fluorescence spectra were measured at an excitation wavelength of 387 nm and normalized to the fluorescence intensity of BADAN attached to the GGBP/H152C apoform. The BADAN excitation spectra measured at fluorescence recording wavelengths of 475 and 530 nm and normalized to the intensity at the maximum of the spectrum are shown by blue dashed curves. The anisotropy spectra of BADAN fluorescence were measured at an excitation wavelength of 387 nm and are represented by red symbols. The excitation anisotropy spectra of BADAN were measured at a fluorescence recording wavelength of 530 nm and are represented by blue symbols.

The position and shape of the BADAN fluorescence excitation spectrum do not match those of the absorption spectrum of the dye and significantly depend on the fluorescence recording wavelength (Figure 2). These indicate the heterogeneity of the ensemble of BADAN molecules in the ground state [56,57]. The shape of the excitation anisotropy fluorescence spectrum of BADAN in the long-wavelength absorption band (Figure 2) indicates the collinearity of the absorption and emission dipole moment, which confirms the known data on the absence of reorientation of the dipole moment of the dye molecule upon transition to the excited state [21].

**Figure 3:**
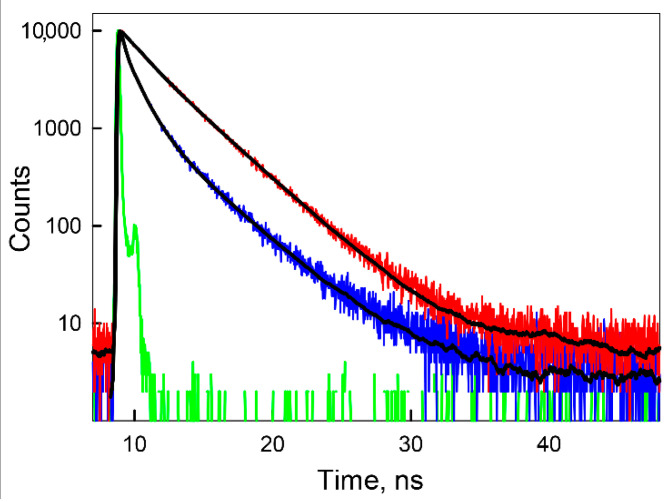
The fluorescence decay of BADAN bound to GGBP/H152C in apo (blue curve) and holoform (red curve) in semilogariphmic scale. The green curve represents the instrument response function. The best fits of BADAN decay are represented by black curves. The excitation wavelength was 372 nm.

The fluorescence decay of BADAN linked to apoform of GGBP/H152C (Figure 3) in the framework of the three-exponential model is characterized by decay times of 3.59 ns, 1.13 ns, and 0.32 ns and the rms lifetime of the excited state, <*τ*>, equal to 1.35 ns ($\bar{\tau }$ = 1.01 ns) (Table S1). 

**Figure 4:**
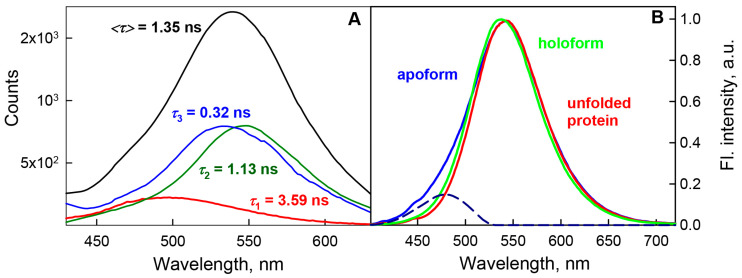
Decomposition of the fluorescence spectrum of BADAN linked to the GGBP/H152C apoform. Panel (**A**) Decay-associated spectrum of BADAN linked to GGBP/H152C apoform after global analysis of 100 dye decay curves recorded from 430 to 630 nm with step 2 nm. Colors indicate spectra associated with the particular decay time. Excitation wavelength was 372 nm. Panel (**B**) Decomposition of the fluorescence spectrum of BADAN linked to the GGBP/H152C apoform into components based on the analysis of the characteristics of the dye attached to various structural states of GGBP/H152C. The fluorescence spectra of BADAN attached to GGBP/H152C in the apoform, the holoform, and the unfolded state are shown in blue, green, and red solid curves, respectively. The spectra are normalized to the fluorescence intensity at their maximum. The short-wavelength component of BADAN fluorescence obtained by subtracting the fluorescence spectrum of the dye attached to denatured GGBP/H152C from the spectrum of the dye attached to the native apoprotein is shown by the blue dashed curve. Excitation wavelength was 387 nm.

The time-resolved fluorescence characteristics of BADAN are indicative of shortwave emission from the longest-lived component of the dye under these conditions (Figure 4A). Previously, the deactivation of the excited state of BADAN under these conditions was defined as biexponential with decay times of 0.5 and 1.3 ns and <*τ*> = 0.8 ns [29].

The multi-exponential decay of the dye fluorescence, as well as the dependence of the position and shape of the dye excitation spectrum, suggest that the population of BADAN molecules in the excited state is heterogeneous. Analyzing the shape of the fluorescence spectra of BADAN attached to GGBP/H152C in solutions with different contents of GdnHCl, we noted the coincidence of the long-wavelength part of the spectrum of the dye linked to the GGBP/H152C apoform with the shape of the long-wavelength part of the GGBP/H152C-BADAN spectrum in 3 M GdnCl (Figure 4B). We assumed that some part of the dye molecules linked to the GGBP/H152C apoform in the absence of denaturants fluoresces from the same state as BADAN molecules attached to the unfolded protein molecules in 3 M GdnHCl. Based on this assumption, we divided the fluorescence spectrum of BADAN linked to the GGBP/H152C apoform into two components: a long-wavelength one with a maximum at about 540 nm, which coincides in shape with the GGBP/H152C-BADAN spectrum in 3 M GdnHCl, and a short-wavelength one with a maximum at about 475 nm (Figure 4B). The short-wavelength component of BADAN fluorescence was determined as shown in Figure 4B. This approach for the separation of the fluorescence spectra of BADAN attached to different GGBP mutants into red and blue components was used in all studied conditions.

Previously, spectral characteristics of BADAN, localized in various regions of the lipid-water interface, were analyzed using the addition of a dye to various mutant forms of the membrane protein M13 [20,21]. In the fluorescence spectrum of BADAN in a polar environment, the PICT and TICT states are responsible for short-wavelength (λmax = 476 nm) and long-wavelength (λmax = 544 nm) components, respectively [20]. The authors of this work assume that the PICT state of the dye is formed almost immediately after the absorption of a light quantum by the BADAN molecule, i.e., the lifetime of the locally excited (LE) state is negligible and the LE state of BADAN does not participate in the fluorescence of this dye. While the TICT state is an equilibrium excited state of the dye that appears upon relaxation of the PICT state of the dye in a polar environment, or from the solvated ground state [20].

Our results obtained for the GGBP/H152C apoform are close to the characteristics of the BADAN fluorescence components obtained in [20]. Within the framework of this model, it can be assumed that the long-wavelength component of BADAN fluorescence attached to the GGBP/H152C apoform is determined by emission from the solvated TICT state of the dye, and the short-wavelength component of BADAN fluorescence is due to emission from the unsolvated PICT state. Analysis of the time-resolved spectrum of BADAN indicates that the state responsible for the emission of the short-wavelength component of BADAN fluorescence has the longest decay time of all dye fluorescence components under these conditions (Figure 4A). Accordingly, the long decay time of the short-wavelength component of BADAN fluorescence may be due to the absence of solvation of dye molecules that fluoresce from the PICT state. In this case, the dependence of the position and shape of the fluorescence excitation spectrum of BADAN attached to the GGBP/H152C apoform on the fluorescence recording wavelength suggests that the heterogeneity of the ensemble of fluorescent dye molecules is caused not only by the existence of several excited states of BADAN, which are formed as a result of relaxation of the PICT state of the dye after absorption, but also by the presence of BADAN fluorescent isomers in the solution.

The interaction of GGBP/H152C with glucose causes a threefold increase in the BADAN fluorescence intensity, makes the dye’s fluorescence spectrum narrower, and causes its short-wavelength shift by 4–5 nm (Figure 2). The complexation of the protein with the ligand is also accompanied by a significant increase in the anisotropy of the dye fluorescence and the lifetime of the excited state of BADAN. Deactivation of the excited state of BADAN attached to the GGBP/H152C holoform obeys the biexponential law with <*τ*> = 3.09 ns ($\bar{\tau }$= 2.78 ns) and decay times of 3.39 ns and 1.33 ns (Table S1). Our results agree with the earlier one obtained in the framework of the monoexponential model with = 3.1 ns [29]. Analysis of the time dependences of the fluorescence anisotropy of BADAN attached to GGBP/H152C showed that the complex formation of GGBP/H152C with glucose leads to a decrease in the amplitude of high-frequency torsional vibrations of the dye molecule (Table S2).

The comparison of the fluorescence spectra of BADAN linked to holoform of GGBP/H152C in native and denaturing conditions (3 M GdnHCl) conditions showed that they have the same shape, but the spectrum recorded in the native condition is blue-shifted (3–4 nm) in comparison with that in 3 M GdnHCl (Figure 4B). This suggested that the dye linked to the GGBP/H152C holoform emits from the TICT solvated state. However, the stationary and time-resolved fluorescence characteristics of BADAN indicate that the disappearance of the short-wavelength component of fluorescence from the fluorescence spectrum of BADAN upon the transition of GGBP/H152C from apo to the holoform is accompanied by an increase in the atomic packing density and a slight decrease in the polarity of the BADAN microenvironment, i.e., not due to dye solvation (Figure 2, Table S1). As already mentioned, the active center of GGBP contains a large number of polar residues; therefore, when glucose is inserted into the active center of the protein, the BADAN microenvironment can remain polar, but, owing to the displacement of water molecules by the ligand, it is unsolvated. Considering that the fluorescence decay times of BADAN linked to the GGBP/H152C holoform and the short-wavelength component of the fluorescence of the dye linked to the protein apoform are close, the assumption that the fluorescence of BADAN linked to the GGBP/H152C complex with glucose originates from non-solvated PICT state in the polar environment is more plausible. Within the framework of this hypothesis, dye desolvation may be one of the possible reasons for a threefold increase in the BADAN fluorescence intensity upon the interaction of GGBP/H152C with glucose.

#### 2.1.2. GGBP/W284C Apo- and Holoforms

**Figure 5:**
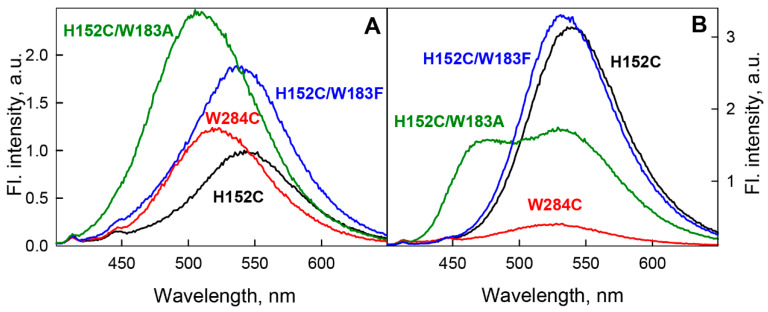
Influence of microenvironmental properties on the fluorescence characteristics of BADAN coupled to various mutant forms of GGBP in the apoform (panel (**A**)) and in complex with glucose (panel (**B**)). The black, red, blue, and green curves correspond to the spectra of the dye attached to GGBP/H152C, GGBP/W284C, GGBP/H152C/W183F, and GGBP/H152C/W183A, respectively. The BADAN fluorescence intensity is normalized to the fluorescence intensity of the dye attached to the GGBP/H152C apoform. The excitation wavelength was 387 nm.

In order to show the effect of the high polarity of the BADAN environment on its fluorescence properties, we examined the fluorescence characteristics of the dye linked to the mutant form GGBP/W284C. BADAN linked to GGBP/W284C is localized in the *N*-terminal domain of GGBP and is much more accessible to the solvent as compared with the dye linked to GGBP/H152C [55]. It was shown that the fluorescence spectrum of BADAN linked to the GGBP/W284C apoform is 20 nm blue-shifted and its fluorescence intensity is 1.2 times higher than that of BADAN linked to GGBP/H152C (Figure 5A). The rate of deactivation of the excited state of BADAN linked to GGBP/W284C is <*τ*> = 2.4 ns, while that for GGBP/H152C-BADAN is 1.35 ns (Table S1). In this case, the interaction of the GGBP/W284C apoform with glucose, accompanied by the inclusion of the nitrogen atom of Lys 263 in the nearest environment of the residue 284 [55], causes a significant decrease in the BADAN fluorescence intensity and a long-wavelength shift of the dye spectrum (Figure 5B). These data indicate that, firstly, the fluorescence characteristics of BADAN linked to the GGBP/H152C apoform are primarily determined by the properties of the amino acid residues included in the dye environment. Second, the introduction of an electron acceptor into the BADAN microenvironment causes quenching of the dye fluorescence.

#### 2.1.3. GGBP/H152C/W183F and GGBP/H152C/W183A Apo- and Holoforms

In addition to BADAN solvation, a possible reason for the low fluorescence intensity of the dye attached to the GGBP/H152C apoform may be the quenching of its fluorescence by the tryptophan residue at position 183. It is known that tryptophan can act as a quencher of BADAN fluorescence in polar solvents owing to electron transfer from the excited state of BADAN to the tryptophan molecule [44]. The distance between the dye linked to native GGBP/H152C apoform and Trp 183 of this protein is less than 10 Å [55], which provides conditions for BADAN fluorescence quenching by this Trp 183 [44]. To check this assumption, we examined the fluorescence of BADAN linked to created mutant forms GGBP/H152C/W183F and GGBP/H152C/W183A.

Substitution of W183F in the active site of GGBP/H152C apoform causes an almost twofold increase in BADAN fluorescence intensity (Figure 5B), while the position and shape of its spectrum practically do not differ from those of BADAN linked to GGBP/H152C. These data indicate that the W183F substitution reduces the quenching of BADAN fluorescence and practically unchanged polarity of the dye environment.

Interestingly, BADAN fluorescence linked to holoforms of GGBP/H152C/W183F and GGBP/H152C is practically the same (Figure 5B). Taking into account that the mutual position and orientation of the 183 and 152 residues practically does not change upon glucose interaction to GGBP [55], these data indicate the suppression of electron transfer from the excited BADAN molecule to Trp 183 in holoprotein owing to stacking interactions between glucose and tryptophan 183.

**Figure 6:**
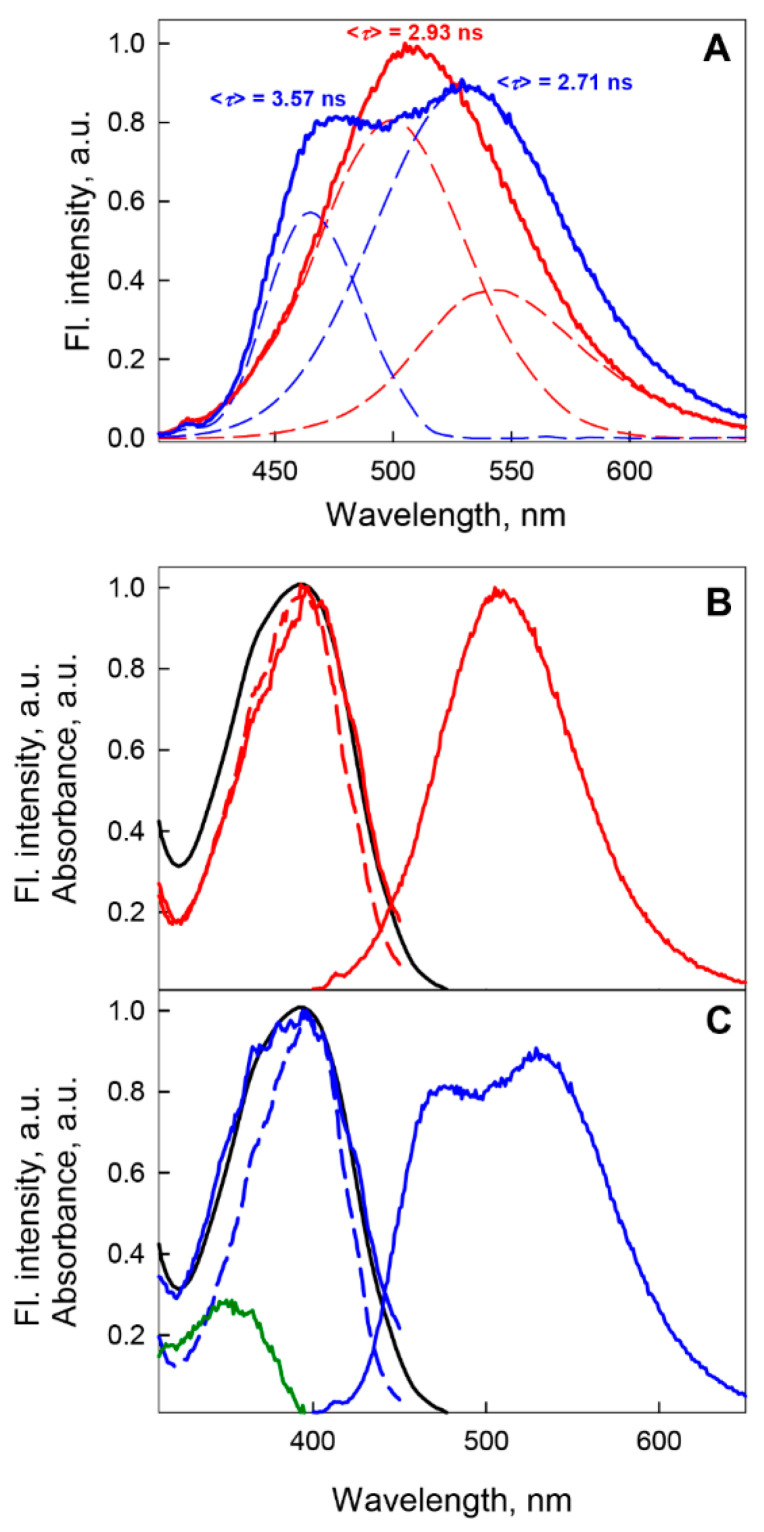
Characteristics of BADAN linked to GGBP/H152C/W183A in apo (red curves) and holo (blue curves) form. Panel (**A**) The fluorescence spectra of BADAN linked to GGBP/H152C/W183A (solid curves) and their decomposition into components (dashed curves); the excitation wavelength was 387 nm. Panels (**B**,**C**) The excitation spectra of fluorescence recorded at 475 nm (dashed curves) and 540 nm (solid curves). The result of subtraction of the excitation spectrum recorded at 475 nm from that recorded at 540 nm is shown by the green curve panel (**C**). Absorption spectrum of BADAN linked to GGBP/H152C/W183A is presented by the black curve.

The introduction of a nonaromatic nonpolar residue into the immediate environment of BADAN by the change W183A leads to a blue shift of the BADAN fluorescence spectrum by 30 nm relative to the fluorescence spectrum of the dye attached to the GGBP/H152C apoform and an increase in the fluorescence intensity of BADAN linked to GGBP/H152C/W183A by 2.5 times compared with the fluorescence intensity of the dye in GGBP/H152C-BADAN (Figure 5B). Simultaneously (in parallel), the root-mean-square lifetime of the excited state of the dye increases more than twofold and amounts to <*τ*> = 2.93 ns (Figure 6). These results also confirm the high polarity of the BADAN environment linked to the GGBP/H152C apoform and the quenching effect of Trp 183 on the dye fluorescence.

Complex formation of GGBP/H152C/W183A with glucose is accompanied by a decrease in the BADAN fluorescence intensity compared with that of apoform and the appearance of two maxima in the fluorescence spectrum: approximately 475 and 530 nm (Figure 6A). To explain this effect, we analyzed the excitation spectra of the dye recorded at different wavelengths of fluorescence. The fluorescence excitation spectrum of BADAN linked to GGBP/H152C/W183A apoform and recorded at 540 nm (red edge of fluorescence spectrum) coincides with the red band of the BADAN absorption spectrum (Figure 6B), while the fluorescence excitation spectrum recorded at 475 nm (blue edge of fluorescence spectrum) does not coincide with the long-wavelength band of the absorption spectrum of the dye and is narrower (Figure 6B).

Thus, for BADAN linked to the GGBP/H152C/W183A holoform, the excitation spectrum recorded at 540 nm is a broad band, including that recorded at 475 nm (Figure 6C). This means that BADAN molecules linked to the GGBP/H152C/W183A glucose complex and emitting in the short-wavelength part of the spectrum can also fluoresce in the long-wavelength region. In turn, this indicates a decrease in the energy of the excited state of such dye molecules owing to their relaxation under these conditions, i.e., about the transformation of the PICT state of BADAN into TICT.

The comparison of the excitation fluorescence spectra of BADAN linked to the holoform and apoform of GGBP/H152C/W183A recorded at 475 and 540 nm revealed a spectral band with a maximum at about 350 nm, corresponding to the absorption of BADAN linked to holoform of molecules. We have previously shown the existence of a mechanism for non-radiative deactivation of the excited state of BADAN, which is associated with the rotation of the fragments of the dye molecule relative to each other [36]. Apparently, during the transition of GGBP/H152C/W183A from apo to the holoform, alanine 183 and some BADAN molecules are oriented in such a way that this causes deformation of the BADAN structure and restriction of the mobility of fragments of the dye molecule relative to each other. In turn, this causes the transformation of the non-radiative deactivation channel of the excited state of such BADAN molecules into a radiative one.

Complex formation of GGBP/H152C/W183A with glucose is accompanied by a short-wavelength shift of both BADAN fluorescence components, an increase in the contribution of the long-wavelength component to the total dye fluorescence intensity, and a slight decrease in the total BADAN fluorescence intensity (Figure 5A). The data obtained indicate an increase in the fraction of relaxed BADAN molecules emitting from the TICT state, with a simultaneous decrease in the polarity of the solution. Apparently, some dye molecules that were non-emitting, being bound to GGBP/H152C/W183A in apoform, contribute to fluorescence in the long-wavelength region of the spectrum when bound to holoform of protein. An insignificant change in the BADAN fluorescence intensity during the complex formation of GGBP/H152C/W183A with glucose indicates the absence of a significant effect of the interaction of GGBP-like proteins with glucose on the fluorescence intensity of BADAN, localized in the active center of such proteins, in the absence of aromatic residues in the immediate environment of the dye, which confirms our hypothesis about the violation of the conditions for quenching BADAN fluorescence by tryptophan residue 183 during stacking interactions between this residue and the glucose molecule.

The time-resolved characteristics of BADAN attached to the GGBP/H152C/W183A complex with glucose, obtained by recording fluorescence in the blue and red regions of the spectrum, differ significantly. The BADAN fluorescence decay curve at 475 nm obeys a biexponential law with <*τ*> = 3.57 ns and decay times of 3.8 and 0.6 ns, respectively (Figure 6A). The dye fluorescence decay at 540 nm is characterized by <*τ*> = 2.71 ns and decay times of 3.56, 1.52, and 0.33 ns (Figure 6A). The data obtained indicate that dye molecules with decay times of 3.5–3.8 ns and 0.3–0.6 ns can fluoresce both in the blue and red parts of the spectrum, which is in agreement with the data obtained by analyzing the stationary fluorescence characteristics of BADAN. The red component of BADAN fluorescence with a decay time of 1.52 ns, also observed upon deactivation of the dye attached to the GGBP/H152C/W183A apoform, can apparently be related to the relaxed TICT state of BADAN.

### 2.2. C. Hanging of BADAN Fluorescent Characteristics with Unfolding of GGBP Mutants

#### 2.2.1. Unfolding of GGBP/H152C in Ligand-Free and Ligand-Bound States

**Figure 7:**
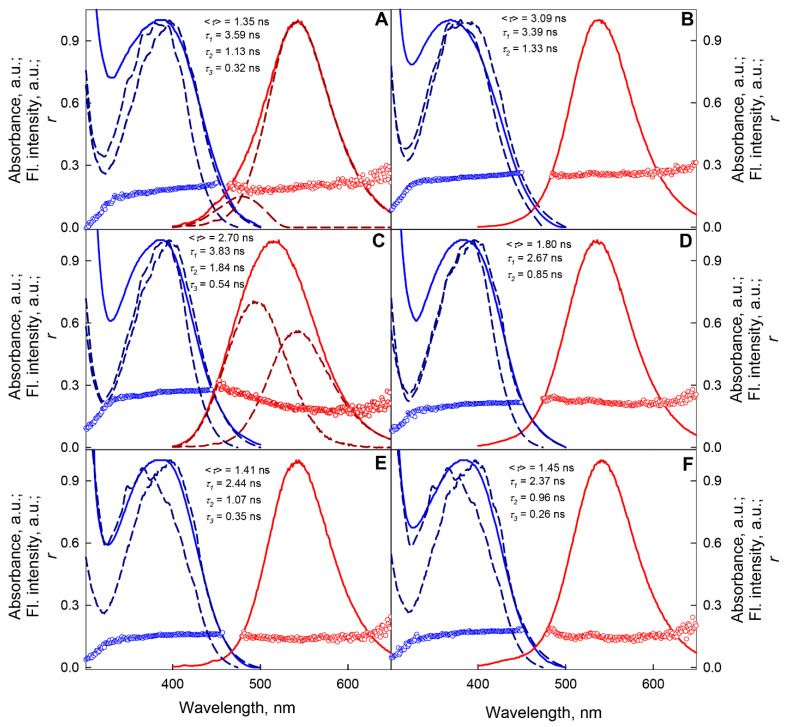
GdnHCl-induced changes in the spectral characteristics of BADAN linked to GGBP/H152C apo- (**left panels**) and holoform (**right panels**). The characteristics of the dye linked to the native protein. The characteristics of the dye linked to the native protein (Panels (**A**,**B**)), partially folded state of apo-protein in 0.5 M GdnHCl (Panel (**C**)), partially folded state of holo-protein in 1.5 M GdnHCl (Panel (**D**)), and unfolded protein in 3M GdnHCl (Panels (**E**,**F**))are presented. The BADAN fluorescence spectra, components of the fluorescence spectra, and fluorescence anisotropy spectra at λ*_ex_* = 387 nm are indicated by red solid and dashed curves and red symbols, respectively. The BADAN absorption spectra, excitation spectra recorded at 475 and 530 nm, and excitation anisotropy spectra recorded at 530 nm are shown by blue solid curves and dashed curves and blue symbols, respectively.

**Figure 8:**
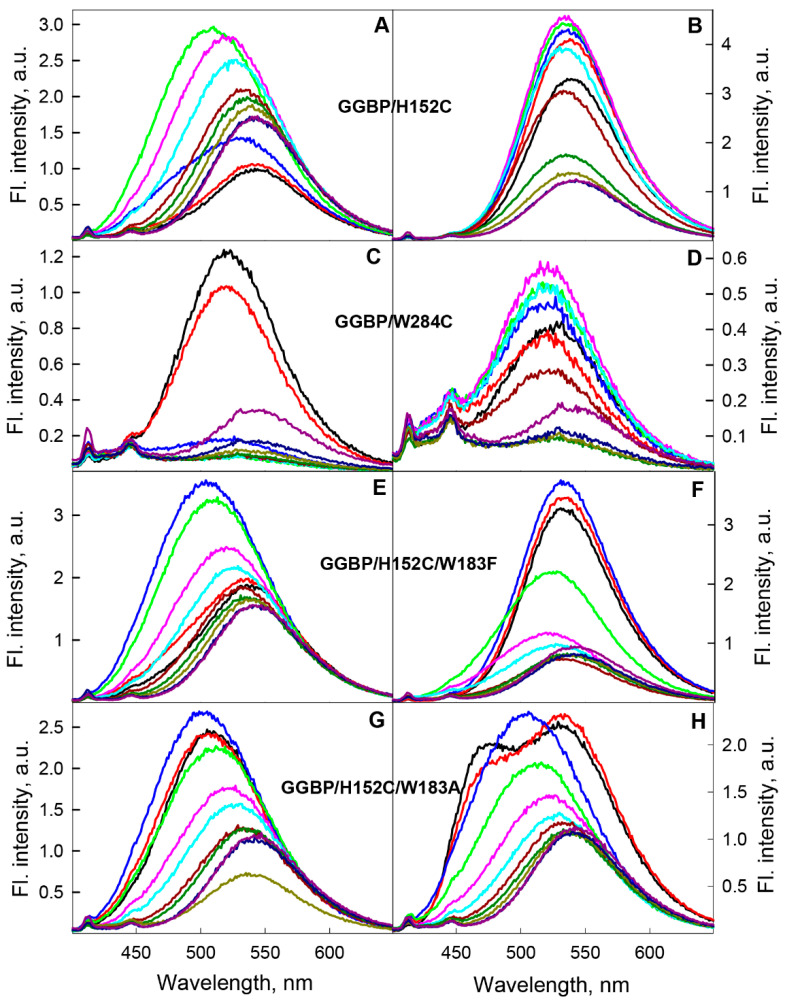
Fluorescence spectra of BADAN linked to mutant forms of GGBP in apo (**left panels**) and holoform (**right panels**) in GdnHCl solutions. Panels (**A**–**H**) Spectra of GGBP/H152C, GGBP/W284C, GGBP/H152C, GGBP/H152C/W183F, and GGBP/H152C/W183A. Black, red, blue, green, pink, blue, dark red, dark green, dark yellow, dark blue, and violet curves characterize the BADAN fluorescence spectra in solutions containing 0, 0.1, 0.3, 0.5, 0.7, 0.9, 1.2, 1.5, 2, 3, and 4 M GdnHCl, respectively. The fluorescence intensity of BADAN under all experimental conditions was normalized to the fluorescence intensity of the dye attached to the GGBP/H152C apoform. The excitation wavelength was 387 nm.

In the range of pre-denaturation concentrations of GdnHCl (from 0 to 0.2 M) and urea (from 0 to 0.5 M), in which, according to intrinsic UV fluorescence data, the protein retains its native structure, the fluorescent characteristics of BADAN attached to GGBP/H152C apoform are practically the same (Figures S1 and S2). In the concentration range from 0.2 M to 0.5 M GdnHCl and from 0.5 M to 1.5 M urea, a redistribution of the contribution of the blue and red components to the total fluorescence intensity of BADAN, the increase in the fluorescence intensity, the lifetime of the excited state, and the contribution of the long-lived component to the total BADAN fluorescence are observed (Figure 7 and Figures S1–S4, Table S1). An increase in the total fluorescence intensity indicates a decrease in the dye fluorescence quenching due to a violation of the conditions for effective BADAN fluorescence quenching by Trp 183. It should be noted that the redistribution of the contribution of the blue and red components to the total fluorescence intensity of the dye in these ranges of denaturant concentrations is not associated with a violation of the conditions of BADAN fluorescence quenching by Trp 183, as it is known that electron transfer from BADAN and tryptophan molecules is not accompanied by a change in the shape of the dye spectrum [44]. The steady state and time-resolved characteristics of the anisotropy of the dye fluorescence indicate the absence of a significant change in the packing density of the atoms in the microenvironment of BADAN linked to the GGBP/H152C apoform in this denaturant concentrations (Figure 7, Table S2). The steady state and time-resolved fluorescence characteristics of BADAN linked to the GGBP/H152C apoform are close to those of the dye linked to the GGBP/H152C/W183A apoform (Figure 8). This means that the dye linked to the GGBP/H152C apoform in solutions with about 0.5 M GdnHCl is in a substantially non-polar environment and emits predominantly from the PICT state.

Correlation of the BADAN fluorescence linked to GGBP/H152C with the intrinsic UV fluorescence of GGBP/H152C, indicating the loss of the structure of the C-terminal domain of the protein at 0.2–0.5 M GdnHCl and at 0.5–1.5 M urea, argues for the appearance of a partially unfolded state at this concentration of denaturants (Figure S1). Apparently, the formation of this state is due to the unfolding of the C-terminal domain of GGBP/H152C, while the *N*-terminal domain of the protein retains its structure.

A further increase in the concentration of GdnHCl and urea is accompanied by a decrease in the total intensity of BADAN fluorescence and the contribution of the long-lived component to the total fluorescence intensity of the dye, a red shift and narrowing of the fluorescence spectrum, a decrease in the average lifetime of the excited state, and anisotropy of the fluorescence of BADAN (Figure 7 and Figures S2–S4, Table S1). Such changes in the fluorescence characteristics of the dye indicate the quenching of its fluorescence due to the increased BADAN accessibility to the solvent during protein unfolding. At a concentration of GdnHCl (urea) above 2.5 M (4 M) urea, the fluorescence spectrum of BADAN linked to the GGBP/H152C apoform lacks a short-wavelength component, and the dye emits only from the “TICT” state (Figure 8). This suggests that, under these conditions, all BADAN molecules are solvated. At the same time, the fluorescence intensity of the dye linked to unfolded GGBP/H152C is slightly higher than the fluorescence intensity of BADAN linked to the native protein, and the characteristics of the deactivation of the excited state of the dye linked to the protein in the absence of denaturants and at high concentrations of GdnHCl and urea are similar (Figure 7, Table S1). Consequently, the solvation of BADAN exerts a lower quenching effect on its fluorescence than tryptophan residue 183.

#### 2.2.2. Unfolding of GGBP/H152C in Ligand-Free and Ligand-Bound States

At 0–0.8 M GdnHCl, where, according to UV fluorescence, the GGBP/H152C complex with glucose retains its structure, there is a slight increase in fluorescence intensity and anisotropy, a decrease in the amplitude of high-frequency torsional vibrations, and a slight blue shift of fluorescence spectrum of BADAN linked to the holoform of this protein (Figure 7 and Figure 8, Tables S1 and S2). This indicates the compaction of the microenvironment of BADAN linked to the GGBP/H152C holoform in this denaturant concentration range.

The unfolding of the GGBP/H152C holoform is accompanied by a significant decrease in anisotropy, excited state lifetime, and fluorescence intensity, as well as the red shift in the BADAN fluorescence spectrum, which indicates an increase in the accessibility of BADAN to the solvent (Figure 7 and Figure 8, Table S1). In the entire range of used GdnHCl concentrations with GGBP/H152C holoform unfolding, the blue component of BADAN fluorescence was not detected. The dependencies of the fluorescence characteristics of BADAN linked to the GGBP/H152C-Glc complex on the denaturant concentration are a sigmoidal curve and generally correspond to the denaturation curves of the GGBP/H152C holoform obtained based on intrinsic UV fluorescence data. This indicates a one-step unfolding of the GGBP/H152C complex with glucose, which is consistent with the literature data on the preferential stabilization of the C-terminal domain of the protein during the interaction of GGBP with glucose [39,58,59,60,61].

#### 2.2.3. Unfolding of GGBP/W284C in Ligand-Free and Ligand-Bound States

As it is not possible to perform a control experiment to determine the effect of the concentration dependences of GdnHCl and urea on the characteristics of free BADAN because of the significantly different solubility of the dye in the test solutions (BADAN is practically insoluble in water and soluble in GdnHCl and urea), the fluorescence characteristics of BADAN localized on the surface of the *N*-terminal domain of GGBP/W284C in solutions with different concentrations of denaturants were studied. An increase in the denaturant concentration in the range of GdnHCl from 0 to 2 M (urea from 0 to 2.5 M) causes quenching of the dye fluorescence owing to the increased accessibility of BADAN to the solvent as the protein unfolds. A further increase in the concentration of denaturants in solutions containing unfolded protein leads to a slight increase in the fluorescence intensity of the dye and a long-wavelength shift of its spectrum (Figure 8C). The position and shape of the GGBP/W284C-BADAN fluorescence spectrum at high denaturant concentrations coincide with the position and shape of the GGBP/H152C-BADAN spectrum under similar conditions. The increase in the fluorescence intensity of BADAN linked to GGBP/W284C at high denaturant concentrations may be due to several reasons. First, it is known that the aqueous environment has a significant quenching effect on BADAN fluorescence, probably owing to the formation of a hydrogen bond between the BADAN oxygen atom and a water molecule (solvent deprotonation) [29]. GdnHCl and urea can contribute to the structuring of water and a change in the network of hydrogen bonds in the near-surface region of the protein, which in turn can cause a change in the solvation shell of the dye [62,63,64]. Secondly, under these conditions, various amino acid residues may appear in the immediate environment of the dye, as proteins retain structural elements even in solutions with high concentrations of denaturants [65,66,67]. Considering that an increase in the concentration of GdnHCl in solutions containing unfolded GGBP/H152C is also accompanied by a slight increase in the fluorescence intensity of the dye covalently linked to the protein, the first assumption looks more plausible.

As the fluorescence of BADAN linked to the GGBP/W284C complex with glucose is significantly quenched, an increase in the concentration of denaturants in solutions containing the holoform of this protein does not cause significant changes in the fluorescent characteristics of the dye. In the concentration range of GdnHCl from 0 to 0.7 M, there is a slight increase in the fluorescence intensity of BADAN linked to the GGBP/W284C holoform (Figure 8D). Apparently, this is caused by the stabilizing effect of low concentrations of denaturant on the structure of the GGBP/W284C complex with glucose (Figure S5) [37].

The unfolding of the GGBP/W284C complex with glucose is accompanied by quenching of the dye fluorescence. In the range of GdnHCl concentrations above 2 M, the fluorescence characteristics of BADAN linked to the GGBP/W284C holoform change similarly to the characteristics of the dye linked to the apoform of this protein.

The data obtained allow us to conclude that an increase in the accessibility of the dye to solvent molecules as the protein is denatured promotes quenching of BADAN fluorescence until the GdnHCl/urea content in the solution changes the properties and structure of the solvent.

#### 2.2.4. Unfolding of GGBP/H152C/W183F and GGBP/H152C/W183A Mutant Forms in Ligand-Free and Ligand-Bound States

The change in the fluorescence characteristics of the dye linked to the GGBP/H152C/W183F apo and holoform in solutions with different concentrations of GdnHCl is generally consistent with the change in the fluorescence characteristics of BADAN linked to GGBP/H152C under the corresponding conditions (corrected for the lower stability of GGBP/H152C/W183F compared with GGBP/H152C) (Figure 8 and Figure S5).

An increase in the GdnHCl concentration up to 0.3 M leads to spectrum broadening, an increase in fluorescence intensity, and a blue shift of the fluorescence spectrum of BADAN linked to GGBP/H152C/W183F apoform (Figure 8E). A further increase in the GdnHCl concentration causes a decrease in the BADAN fluorescence intensity and red shift of the fluorescence spectrum of BADAN linked to the protein (Figure 8E). Moreover, in contrast to GGBP/H152C, the fluorescence intensity of the dye linked to unfolded GGBP/H152C/W183F is lower than the fluorescence intensity of BADAN linked to the native protein. This is another indirect evidence of the strong fluorescence quenching of BADAN linked to the GGBP/H152C apoform by the Trp 183 tryptophan residue.

A slight increase in the fluorescence intensity of BADAN linked to holoform of GGBP/H152C/W183F is observed in the region of pre-denaturation GdnHCl concentrations (Figure 8F). Denaturation of the GGBP/H152C/W183F holoform is accompanied by a sharp decrease in the fluorescence intensity of the dye, which indicates a cooperative one-step unfolding of the GGBP/H152C/W183F holoform. At the same time, at the concentration range of GdnHCl about 0.5 M, an insignificant blue shift of the BADAN fluorescence spectrum is observed (Figure 8F). The spectral characteristics of BADAN linked to holo and apoform of GGBP/H152C/W183F in concentrated solutions of GdnHCl are practically the same. The data obtained indicate that the substitution of Trp 183 with Phe in the active center of GGBP/H152C does not affect significantly the unfolding pathway of the apo and holoforms of the protein. 

The change in the fluorescence characteristics of BADAN linked to the GGBP/H152C/W183A apoform induced by GdnHCl in general resembles that of the dye linked to the GGBP/H152C apoform (Figure 8G). However, a slight increase in the fluorescence intensity and a slight blue shift of the fluorescence spectrum of BADAN linked to the GGBP/H152C/W183A apoform at 0–0.3 M GdnHCl, corresponding to the unfolding region of the C-terminal domain of this protein, contrasts with a significant increase in the fluorescence intensity and a significant blue shift of the fluorescence spectrum of BADAN linked to the GGBP/H152C at the same conditions (Figure 8G). These differences are due to the amino acid substituttion W183A, which makes the microenvironment of the dye non-polar and eliminates quenching of BADAN fluorescence. A further increase in the GdnHCl concentration causes a decrease in the BADAN fluorescence intensity and a red shift of the dye fluorescence spectrum, which indicates a sharp increase in the dye’s accessibility to the solvent upon denaturation of its *N*-terminal domain. The fluorescence characteristics of BADAN linked to unfolded GGBP/H152C/W183A are identical to those of the dye linked to the GGBP/H152C/W183F and GGBP/H152C mutants in the denatured state.

The fluorescence characteristics of BADAN bound to holoform of GGBP/H152C/W183A with glucose and the fluorescence characteristics of the dye bound to the GGBP/H152C holoform undergo completely different changes in GdnHCl solutions. In the range of 0–0.3 M GdnHCl, the fluorescence spectrum of BADAN linked to the complex of GGBP/H152C/W183A with glucose becomes narrower and undergoes a blue shift, as a result of which the fluorescence characteristics of the dye become close to those of BADAN linked to the protein apoform (Figure 8H). This indicates a significant change in the properties of the dye microenvironment, which results in the loss of fluorescence of the band with an absorption maximum at about 350 nm. A further increase in the denaturant concentration causes a decrease in the fluorescence intensity of BADAN and a red shift in its fluorescence spectrum, similar to that of BADAN linked to the GGBP/H152C/W183A apoform (Figure 8H). The data obtained support a two-stage unfolding of the complex of GGBP/H152C/W183A with glucose in contrast to the holoforms of other studied mutant forms of GGBP. This is consistent with UV fluorescence data, indicating an almost complete absence of the GGBP/H152C/W183A structure stabilization by glucose (Figure S5).

## 3. Conclusions

**Figure 9:**
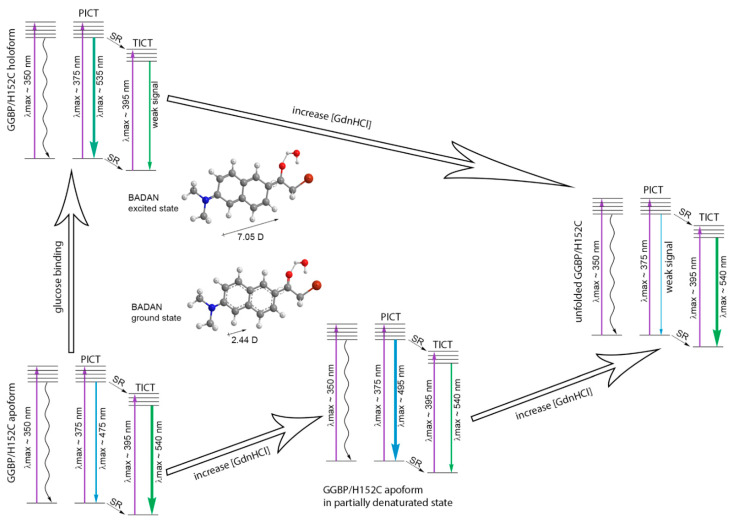
Diagram illustrating BADAN photoprocesses during conformational changes in GGBP/H152C during the interaction of this protein with glucose and the unfolding of the GGBP/H152C apo and holoform induced by GdnHCl. The energy levels of the dye are indicated by horizontal lines, vertical purple arrows illustrate the transition of BADAN molecules from the ground to an excited state, blue and green arrows illustrate the radiative transitions of BADAN from PICT (planar intramolecular charge transfer state) and TICT (twisted intramolecular charge transfer state) to the ground state, and wavy arrows indicate nonradiative BADAN transitions. The relaxation of the excited and ground state of the dye under the action of the solvent is indicated by the abbreviation SR and is represented by dashed arrows.

Analysis of the fluorescence characteristics of BADAN linked to the mutant forms GGBP/H152C, GGBP/H152C/W183F, GGBP/H152C/W183A, and GGBP/W284C during their unfolding in the absence and in the presence of glucose shed light on the photophysical features of BADAN and allowed to propose a scheme of BADAN photoprocesses that occur during conformational changes in GGBP/H152C (Figure 9). It was found that the fluorescence characteristics of BADAN are determined by the existence of three isomers of the dye in the ground state and two in the excited state. Non-fluorescent BADAN isomers absorb in the short-wavelength part of the long-wavelength band of the spectrum (absorption maximum at 350 nm). The absorption band with a maximum at 385–390 nm is responsible for the absorption of dye isomers, which fluoresce in both the short-wavelength and long-wavelength regions of the BADAN fluorescence spectrum. Dye molecules that absorb light in this band and emit in the short-wavelength part of the fluorescence spectrum are characterized by long decay times (about 3 ns) and a maximum of the fluorescence spectrum in the range 475–530 nm (depending on the polarity of the dye environment). Presumably, these molecules have a plane conformation and emit from the PICT state. Relaxation of such BADAN molecules in an excited state upon their interaction with polar molecules of the dye microenvironment causes the transition to a nonplanar “TICT” state. This causes a significant decrease in the decay times of the dye fluorescence from 3 ns to values of 0.3–1 ns and a red shift of the fluorescence spectrum to the region of about 540 nm. The relaxation of the PICT state of the dye upon its solvation is accompanied by a significant quenching of the dye fluorescence and the formation of a hydrogen bond between the solvent molecules and BADAN. The transition to the TICT state also occurs directly from the solvated ground state bypassing the PICT state.

It was shown that, in addition to solvation, aromatic amino acid residues have a significant quenching effect on BADAN fluorescence. This effect is based on the transfer of an electron from an excited dye molecule to an aromatic amino acid molecule. It was found that stacking interactions between aromatic amino acids and glucose inhibit their quenching of BADAN fluorescence.

BADAN fluorescence is sensitive to the accumulation of partially folded state of the GGBP/H152C apoform, which accumulates with an increase in the GdnHCl concentration in the range of 0–0.5 M because, in this state, the BADAN microenvironment became substantially non-polar and emits from the PICT state. A further increase in the concentration of denaturants in the solutions leads to unfolding of the protein, solvation of dye molecules, and emission of BADAN from the TICT state. The unfolding of the protein holoform, proceeding as an all-or-nothing transition, is accompanied by the solvation of BADAN molecules and the emission of the dye from the TICT state.

## 4. Materials and Methods

### 4.1. Materials

D-glucose, guanidine hydrochloride (GdnHCl) (Sigma, St.Louis, MO, USA), urea, tris(2-carboxyethylphosphine (TCEP) (Sigma, St.Louis, MO, USA), and fluorescent dye BADAN (AnaSpec, Fremont, CA, USA) were used without further purification. To determine the GdnHCl and urea concentrations, we relied on the measurement of the refraction coefficient using Abbe refractometer (LOMO, St. Petersburg, Russia).

*E. coli* strain K-12 (*F^+^ mgl503 lacZ lacY* ^+^ *recA1*) carrying an *mglB* gene deletion [68,69] transformed with a pTz18u-*mglB* vector was primary used for obtaining GGBP wild type. Upon induction with D-fructose [70], the expression efficiency of the GGBP protein was rather low. The recombinant protein yield in this system does not exceed 5–8 mg/L of culture. Therefore, for expression, increasing the nucleotide sequence of *mglB* gene was optimized and the gene was recloned into a pET-11d plasmid with the T7 promoter (Stratagene, La Jolla, CA, USA) using *Nco I-BamH I* and *Bgl II* restriction sites. Specific forward and reverse primers were used to insert new restriction sites and a polyhistidine tag at the C-terminal of the gene. Site-directed mutagenesis was performed with the Quik-Change mutagenesis kit (Stratagene, La Jolla, CA, USA) using primers encoding corresponding to amino acid substitutions. Plasmids were isolated from bacterial cells using plasmid DNA isolation kits (Omnix, St. Petersburg, Russia). Primer purification was performed using either reverse-phase chromatography or electrophoresis in a polyacrylamide gel.

pET-11d plasmids encoding for GGBP/H152C, GGBP/W284C, GGBP/H152C/W183A, and GGBP/H152C/W183F mutants were used to transform *E. coli* BL21(DE3) cells. The expression of the proteins was then induced by adding 0.5 mM isopropyl-beta-D-1-thiogalactopyranoside (IPTG; Nacalai Tesque, Kyoto, Japan). Bacterial cells were cultured for 24 h at 37 °C. Recombinant proteins were purified using Ni^++^-agarose packed in His-GraviTrap columns (GE Healthcare, Chicago, IL, USA). Protein purification was controlled using denaturing SDS-electrophoresis in 15% polyacrylamide gel [71].

The labeling of GGBP/H152C and GGBP/W284C with the fluorescent dye BADAN was performed as described by Khan [26] with slight modification. To label proteins with BADAN, 100-fold excess of TCEP was added in solution and then, in the obtained mixture, 10-fold excess of dye was added. Then, the obtained mixture was incubated overnight at 4 °C. Unbound dye was removed by filtration and extensive dialysis against a sodium phosphate buffer.

The experiments were performed in solutions with a protein concentration of 0.2 mg/mL. For the formation of the protein–ligand complex, 20 mM of D-glucose was added to the protein solution. All measurements were made in sodium phosphate buffer solution at pH 7.4. All experiments were performed at 23 °C.

### 4.2. Methods

#### 4.2.1. Steady-State Fluorescence Spectroscopy

The fluorescence experiments were carried out using Cary Eclipse (Agilent, Santa Clara, CA, USA) spectrofluorimeter. The measurements were made at 23 °C with cells 10 × 10 mm (Starna, Atascadero, CA, USA). The fluorescence intensity of BADAN and tryptophan residues was corrected to the primary inner filter effect, as Equation (1) [56]:

(1) $$F_{0}\left(\lambda_{ex}\right)\;=F\left(\lambda_{ex}\right)/W$$

where *W* is a factor that corrects the measured total fluorescence intensity for the so-called primary inner filter effect.

Because the fluorescence measurements were performed using the Cary Eclipse spectrofluorimeter with horizontal slits, the value of the correction factor *W* was calculated based on the following ratio, as Equation (2):

(2) $$W=\frac{\left(1-10^{-A_{\Sigma }}\right)}{A_{\Sigma }}$$

where *A_Σ_* is the total absorbance of exciting light in the solution. Absorption spectra were registered using U-3900H (Hitachi, Tokyo, Japan) spectrophotometer. In [56], it was shown that, corrected in such a manner, the value of the total fluorescence intensity is proportional to the product of the absorbance AFL to the quantum yield of fluorescence q when there is one fluorescent substance in solution.

The excitation wavelength for the intrinsic protein fluorescence was 297 nm. The emission wavelengths for the intrinsic protein fluorescence were 320, 340, and 365 nm. The dye fluorescence was excited at 387 nm. The emission wavelength for the BADAN fluorescence ranged from 400 to 650 nm. The position and form of the UV fluorescence spectra were characterized by the parameter *A* = *F*_320_/*F*_365_, where *F*_320_ and *F*_365_ are the fluorescence intensities measured at emission wavelengths of 320 and 365 nm, respectively [72]. The position and form of the BADAN fluorescence spectra were characterized by the parameter *B* = *F*_498_/*F*_587_, where *F*_498_ and *F*_587_ are the fluorescence intensities measured at emission wavelengths of 498 and 587 nm, respectively. The values of parameters *A*, *B*, and the fluorescence spectra were corrected using the instrument’s spectral sensitivity.

The excitation fluorescence spectra of GGBP/H152C-BADAN were recorded at an emission wavelength of 530 nm. The excitation wavelength for the BADAN fluorescence ranged from 250 to 500 nm. The excitation spectra were corrected for the spectral sensitivity of the instrument and the primary inner filter effect.

Fluorescence anisotropy was determined as follows Equation (3):

(3) $$r=\frac{\left(I_{V}^{V}-GI_{H}^{V}\right)}{\left(I_{V}^{V}+2GI_{H}^{V}\right)}$$

where $I_{V}^{V}$ and $I_{H}^{V}$ are vertical and horizontal components of fluorescence intensity excited by vertically polarized light, and $G={I_{V}^{V}/I}_{H}^{H}$ is a coefficient that determines the different sensitivity of registering system for vertical and horizontal components of fluorescence intensity.

#### 4.2.2. Time-Resolved Fluorescence Spectroscopy

Time-resolved fluorescence measurements were carried out by time-correlated single-photon counting approach using spectrometer Fluotime 300 (PicoQuant, Berlin, Germany) with Laser Diode Head LDH-375 (λ*_ex_* = 375 nm). Measured fluorescence intensity decays were fit to a multi-exponential model, as Equation (4):

(4) $$F(t)=\int_{-\infty }^{t}IRF\left(ť\right)\sum_{i=1}^{n}\alpha_{i}\cdot \exp\left(-\frac{t-ť}{\tau_{i}}\right)dť$$

where *α_i_* is the amplitude and *τ_i_* is the lifetime of *i_th_* decay component, and *IRF* is the instrument response function. The convolution of the model exponential function with the *IRF* was compared to the experimental data until a satisfactory fit was obtained. The *IRF* was measured using cross-correlation of the excitation and fundamental gate pulse. The FluoFit software (Pico Quant, Berlin, Germany) was used for analysis of decay curves.

To characterize BADAN fluorescence, we used two different average lifetimes; that is, amplitude average lifetime as Equation (5)

(5) $$\bar{\tau }=\sum_{i=1}^{}\alpha_{i}\cdot \tau_{i}$$

and intensity average lifetime as Equation (6)

$$<\tau >=\sum_{i=1}^{}S_{i}\cdot \tau_{i}$$

where

(6) $$S_{i}=\frac{\alpha_{i}\cdot \int_{0}^{\infty }\exp\left(-\frac{t}{\tau_{i}}\right)dt}{\sum_{i=1}^{}\alpha_{i}\cdot \int_{0}^{\infty }\exp\left(-\frac{t}{\tau_{i}}\right)dt}=\frac{\alpha_{i}\cdot \tau_{i}}{\sum_{i=1}\alpha_{i}\cdot \tau_{i}}=\alpha_{i}\cdot \frac{\tau_{i}}{\bar{\tau }}$$

As he intensity average lifetime is the average lifetime of a collection of different excited state populations, where the lifetime of each population is weighted by the relative contribution of that population to the total fluorescence [73], this parameter is more preferable for the characterization of multicomponent fluorescence than amplitude average lifetime.

The fluorescence anisotropy decay was obtained as Equation (3) using recording $I_{V}^{V}(t)$ and $I_{H}^{V}(t)$ at magic angle conditions. *G* coefficient for Fluotime 300 is equal to 1. The anisotropy decays were fit as a two-time approximation as Equation (7):

(7) $$r(t)=\sum_{i}^{2}r_{i}\cdot e^{-t/\varphi_{i}}$$

where *r_i_* is the anisotropy of *i_th_* component, and *φ_i_* is the rotation correlation time of the *i_th_* component. According to this approximation, a decrease in the fluorescence anisotropy of BADAN linked to GGBP/H152C is a sum of “slow” and “fast” motions of this dye [74]. The “slow” motion corresponds to the rotation of protein as a whole with rotation relaxation time *τ_slow_* = 3 *φ_slow_*, and “fast” motion corresponds to the rotation of dye with rotation relaxation time *τ_fast_* = 3 *φ_fast_*. The anisotropy profiles allow for the calculation of the mean amplitude of dye motions *θ* as follows Equation (8) [75,76]:

(8) $$\frac{r_{fast}}{r_{0}}=1-\frac{\cos^{2}\theta {\left(1+\cos\theta \right)}^{2}}{4}$$

where $r_{0}=r\left(t=0\right)=r_{fast}+r_{slow}$.

## Supplementary Materials

The following are available online at https://www.mdpi.com/article/10.3390/ijms222011113/s1.
