# Supplementary material for: Photophysical Properties of BADAN Revealed in the Study of GGBP Structural Transitions
Source: Int J Mol Sci. 2021 Oct 15;22(20):11113. doi: 10.3390/ijms222011113 (PMC8540541; doi:10.3390/ijms222011113)
Supplement: Supplementary file 1 [file ijms-22-11113-s001.zip › ijms-1363515-supplementary.pdf]

Supplementary information

Photophysical properties of BADAN revealed in the study of GGBP structural transitions

Alexander V. Fonin [1,*], Sergey A. Silonov [1], Iuliia A. Antifeeva [1], Olga V. Stepanenko [1], Olesya V. Stepanenko [1], Anna S. Fefilova [1, 2], Olga I. Povarova [1], Olesya V. Stepanenko [1], Anastasia A. Gavrilova [1], Irina M. Kuznetsova [1,*] and Konstantin K. Turoverov [1,*]

[1] Laboratory of Structural Dynamics, Stability and Folding of Proteins, Institute of Cytology,
Russian Academy of Sciences, 4 Tikhoretsky Ave., 194064 St. Petersburg, Russia;
[2] Research Center for Molecular Mechanisms of Aging and Age-Related Diseases, Moscow Institute of Physics and Technology, 141700 Dolgoprudny, Russia

* Correspondence: alexfonin@incras.ru (A.V.F.); imk@incras.ru (I.M.K.), kkt@incras.ru (K.K.T.);

Table S1. Fluorescent characteristics of free BADAN and BADAN linked to GGBP variants in GdnHCl and urea solutions

GGBP/H152C-BADAN apoform

[Denaturant]	< >, ns	$_1$, ns	S_1, %	$_2$, ns	S_2, %	$_3$, ns	S_3, %	^{2}	λ_{max}, nm	$r^{387}{}_{530}$
0 M	1.35	3.59	14.88	1.13	66.68	0.32	18.43	0.99	540	0.19
0.5 M GdnHCl	2.70	3.83	50.12	1.84	39.83	0.54	10.05	0.99	515	0.17
1.2 M Urea	2.39	3.84	40.48	1.68	46.01	0.48	13.51	0.99	515	0.17
3 M GdnHCl	1.41	2.44	32.57	1.07	52.54	0.35	14.88	0.94	541	0.14
4 M Urea	1.63	2.96	28.98	1.29	55.61	0.4	15.41	1.04	538	0.14
5.5 M GdnHCl	1.36	2.56	24.09	1.17	57.23	0.4	18.67	0.92	543	0.14
6 M Urea	1.55	2.57	36.36	1.12	52.01	0.35	11.63	0.99	540	0.14

GGBP/H152C-BADAN holoform

[Denaturant]	< >, ns	$_1$, ns	S_1, %	$_2$, ns	S_2, %	$_3$, ns	S_3, %	^{2}	λ_{max}, nm	$r^{387}{}_{530}$
0 M	3.09	3.39	85.76	1.33	14.24			1.10	535	0.26
1.5 M GdnHCl	1.80	2.67	52.8	0.85	47.2			1.09	530	0.20
2 M Urea	2.80	3.70	55.34	2.0	38.18	0.52	6.48	1.07	531	0.20
3 M GdnHCl	1.45	2.37	39.1	0.96	52.08	0.26	8.83	1.01	541	0.14
4 M Urea	1.65	2.90	31.14	1.29	51.81	0.43	17.05	1.05	538	0.14
5.5 M GdnHCl	1.39	2.27	38.57	0.94	52.31	0.27	9.13	1.00	543	0.14
6 M Urea	1.54	2.25	43.02	1.16	46.9	0.31	10.08	1.05	540	0.14

GGBP/W284C-BADAN apoform

[Denaturant]	< >, ns	$_1$, ns	S_1, %	$_2$, ns	S_2, %	$_3$, ns	S_3, %	^{2}	λ_{max}, nm	$r^{387}{}_{530}$
0 M	2.39	5.59	17.94	1.99	65.84	0.42	16.21	1.03	521	0.18
5.5 M GdnHCl	2.01	4.73	13.98	1.75	73.67	0.47	12.35	0.97	544	0.14
6 M Urea	1.99	4.01	15.05	2.01	65.21	0.43	19.74	1.09	538	0.14

free BADAN

[Denaturant]	< >, ns	$_1$, ns	S_1, %	$_2$, ns	S_2, %	$_3$, ns	S_3, %	^{2}	λ_{max}, nm	$r^{387}{}_{530}$
0 M	0.92	2.34	16.84	0.85	57.12	0.19	26.04	0.99	530	0.05
8 M GdnHCl	1.43	3.65	19.10	1.20	56.62	0.23	24.28	0.97	519	0.07
8 M urea	1.37	2.21	41.98	1.01	28.30	0.53	29.72	0.99	525	0.07

Table S2. The anisotropy time-resolved characteristics of BADAN linked to GGBP/H152C.

GGBP/H152C-BADAN apoform						
[Denaturant]	r_{fast}	r_{slow}	r_0	, deg.*	fast, ns	slow, ns
0 M	0.08	0.16	0.24	29	0.3	34
0.5 M GdnHCl	0.14	0.14	0.28	38	0.3	30
1.2 M Urea	0.10	0.13	0.23	34	0.3	28
3 M GdnHCl	0.07	0.07	0.14	39	0.3	5.6
4 M Urea	0.07	0.07	0.14	38	0.3	5.8
5.5 M GdnHCl	0.07	0.08	0.15	36	0.4	4.2
6 M Urea	0.07	0.08	0.15	36	0.3	4.5
GGBP/H152C-BADAN holoform						
[Denaturant]	r_{fast}	r_{slow}	r_0	, degree	fast, ns	slow, ns
0 M	0.07	0.19	0.26	25	0.3	31
1.5 M GdnHCl	0.09	0.14	0.23	31	0.3	30
2 M Urea	0.08	0.16	0.24	28	0.3	28
3 M GdnHCl	0.06	0.13	0.19	36	0.3	4.2
4 M Urea	0.07	0.08	0.15	37	0.3	5.3
5.5 M GdnHCl	0.08	0.10	0.18	34	0.3	4.6
6 M Urea	0.07	0.10	0.17	33	0.3	5

* The mean amplitude of dye motions was calculated according eq.5.

Methods

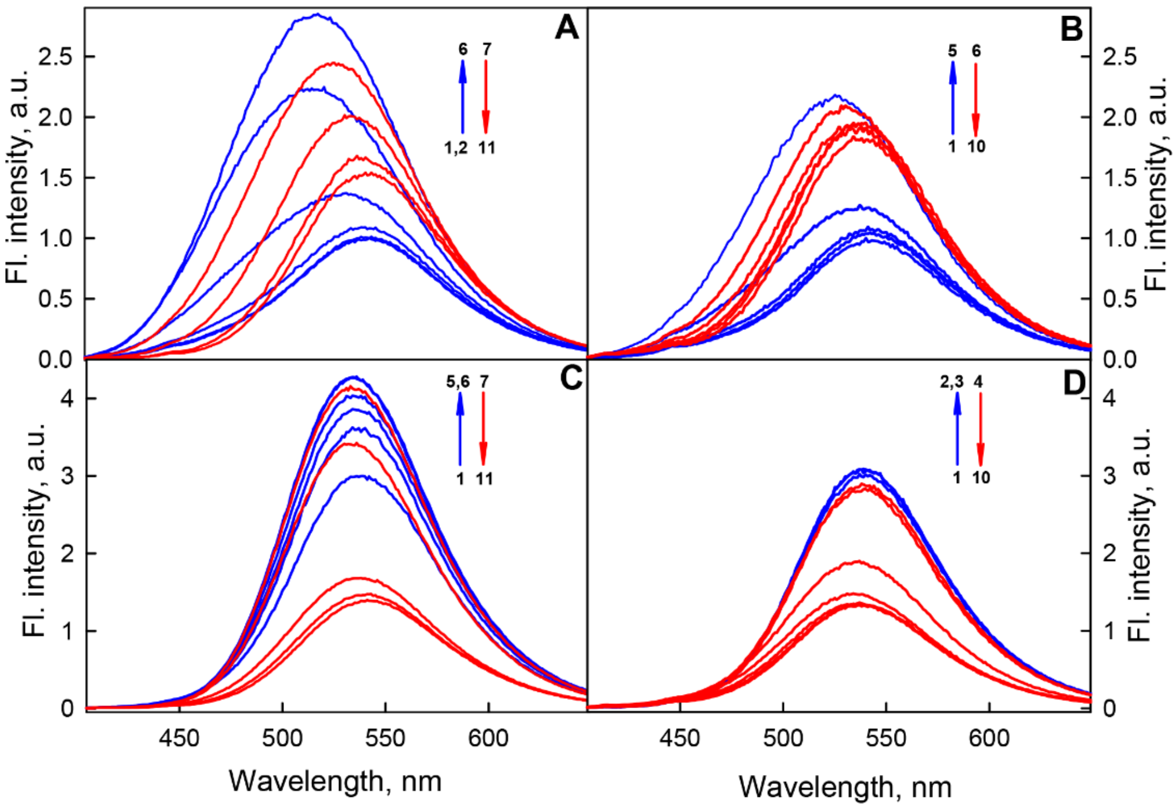

Figure S1. GdnHCl-induced (panels **A**, **C**) and urea-induced (panels **B**, **D**) changing of fluorescent characteristics of BADAN linked to GGBP/H152C in apo (panels **A**, **B**) and holoform (panels **C**, **D**). The GGBP/H152C–BADAN spectra with increasing fluorescence intensity are represented by blue curves, with decreasing fluorescence intensity were represent by red curves. The spectra of GGBP/H152C-BADAN apoform (panel **A**) in the presence of 0, 0.1, 0.2, 0.3, 0.4, 0.6, 0.8, 1.2, 2.3 and 3 M GdnHCl are represented by 1 – 10 curves, respectively. The spectra of GGBP/H152C-BADAN holoform (panel **C**) in the presence of 0, 0.1, 0.2, 0.4, 0.6, 0.8, 1.0, 1.3, 1.8, 2.5, and 3.3 M GdnHCl are represented by 1 – 11 curves, respectively. The spectra of GGBP/H152C-BADAN apo and holoform (panels **B** and **D**) in the presence of 0, 0.1, 0.5, 1.0, 1.5, 2.0, 2.5, 3.0, 3.5 and 4.0 M urea solutions are represented by 1 – 10 curves, respectively. To form protein-ligand complex 20 mM glucose was added in solutions. The excitation wavelength was 387 nm.

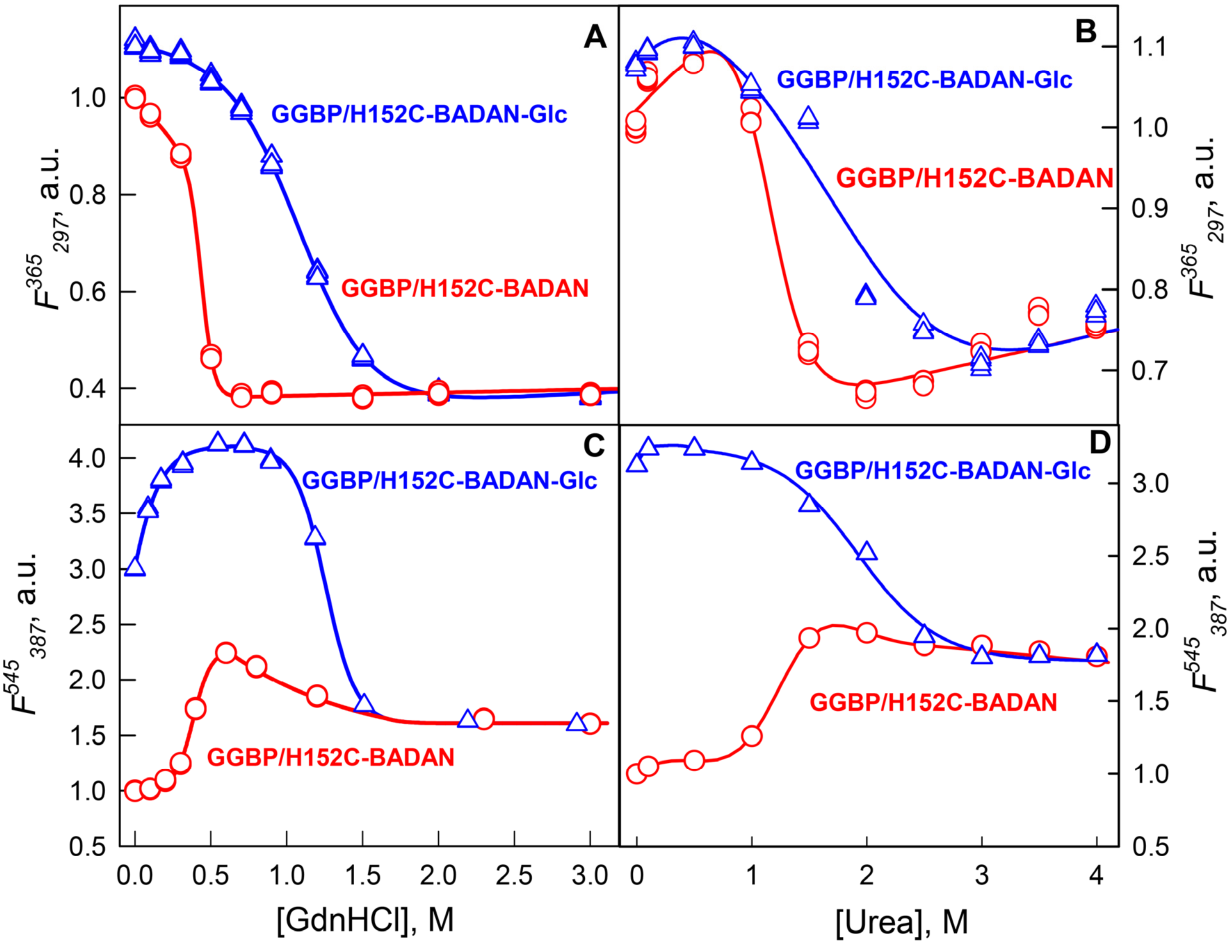

Figure S2. GdnHCl-induced (Panel **A**, **C**) and urea-induced (Panel **B**, **D**) conformational changing of GGBP/H152C–BADAN structure in apo (red curves and circles) and holoforms (blue curves and triangles). Panel **A** and **B** represent the dependence of the $F^{365}{}_{297}$ (fluorescence intensities recorded 365 nm at excitation wavelength 297 nm on the denaturants concentration. Panel **C** and **D** represent the dependence of the fluorescence intensities recorded 587 nm at excitation wavelength 387 nm on the denaturants concentration.

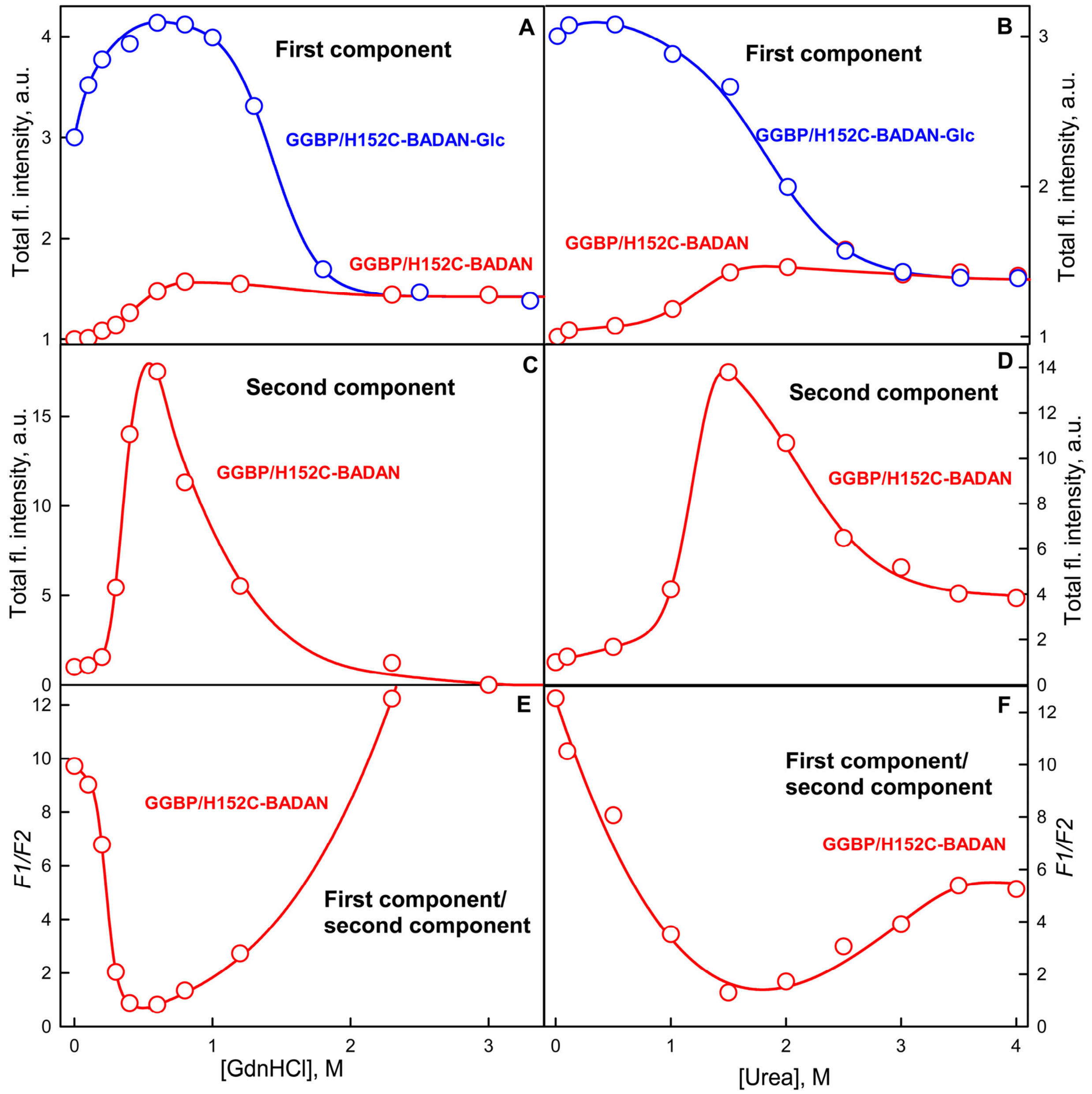

Figure S3. The dependence of fluorescent characteristics of BADAN linked to GGBP/H152C in apo (red curves) and holoforms (blue curves) on GdnHCl (panels **A**, **C**, **E**) and urea (panels **B**, **D**, **F**) concentration. The total fluorescence intensity of long-wave BADAN component represented in Panels **A**, **B**. The total fluorescence intensity of short-wave BADAN component represented in Panels **C**, **D**. The ratio of the total intensity of long-wave BADAN component to the total intensity of short-wave BADAN component was represent in Panels **E**, **F**. The excitation wavelength was 387 nm.

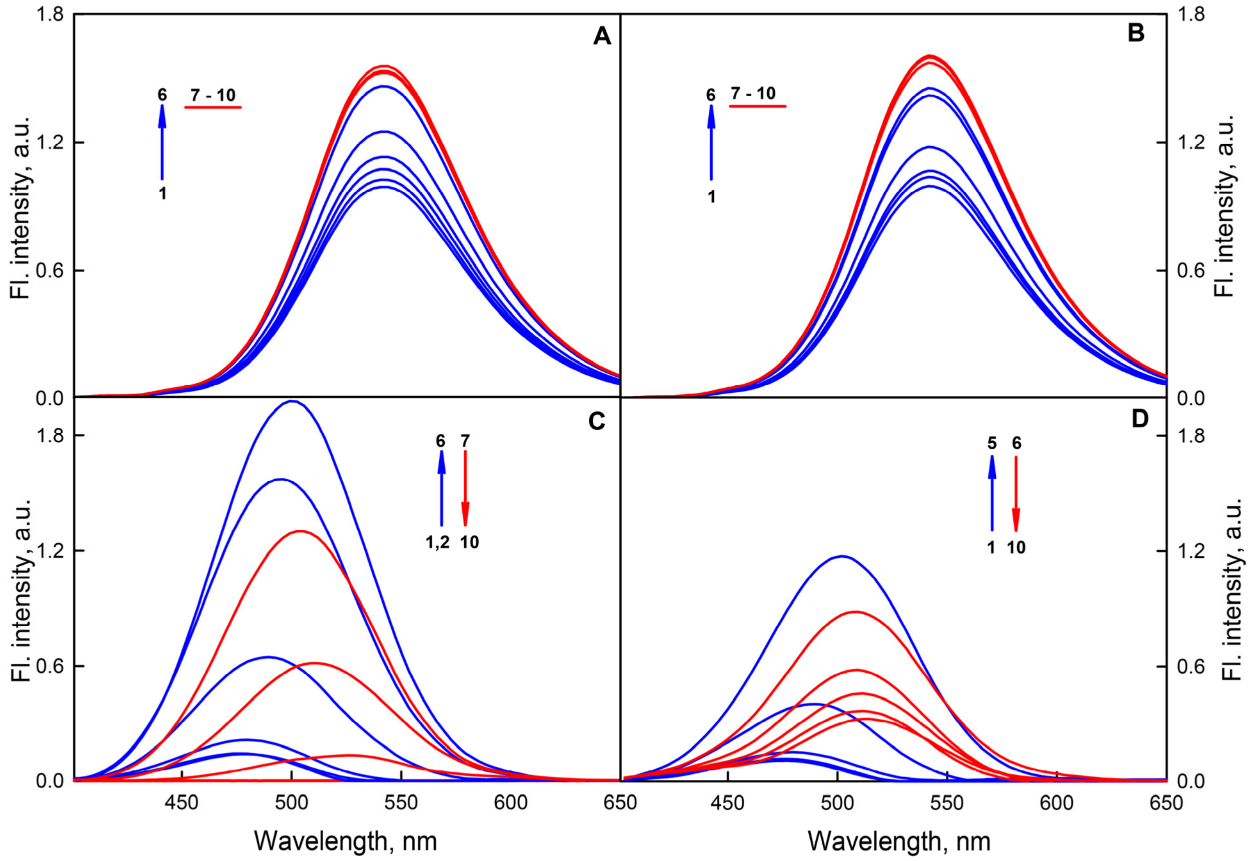

Figure S4. The spectra of long-wave (panels **A**, **B**) and short-wave (panels **C**, **D**) of fluorescence of BADAN linked to GGBP/H152C in GdnHCl (panels **A**, **C**) and urea (panels **B**, **D**). The colors and legends of curves are the same as in Figure 7. The excitation wavelength was 387 nm.

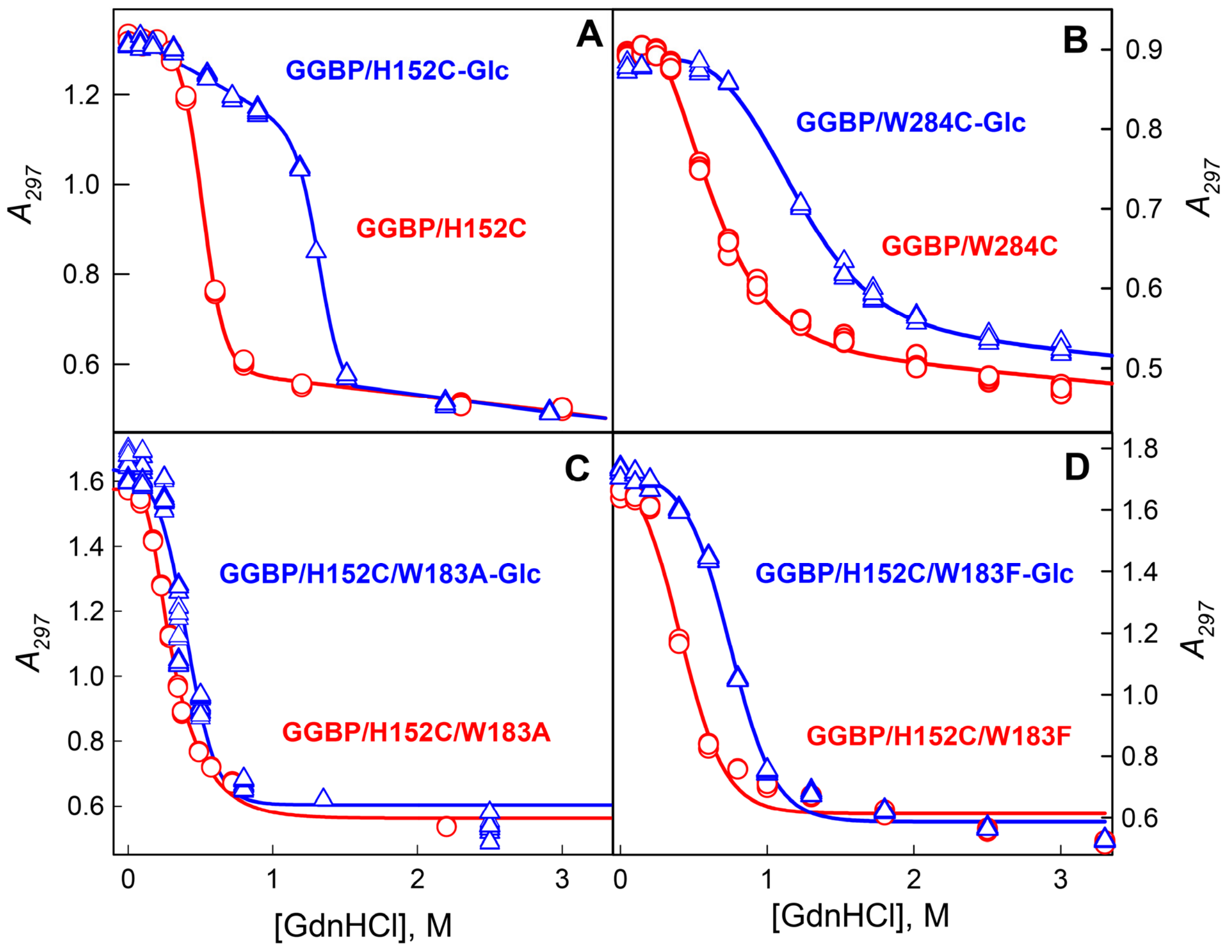

Figure S5. GdnHCl-induced conformational changing of GGBP mutant forms in apo (red curves and circles) and holoforms (blue curves and triangles) according to UV fluorescence data. The dependence of the parameter $A = F^{320}{}_{297}/F^{365}{}_{297}$ ($F^{20}{}_{297}$ and $F^{365}{}_{297}$ are fluorescence intensities recorded at 320 and 365 nm at excitation wavelength 297 nm, respectively) on the GdnHCl concentration for GGBP/H152C, GGBP/W284C, GGBP/H152C/W183A, GGBP/H152C/W183F are represented on panels **A**, **B**, **C**, **D**, respectively.
